# Attitudes to colorectal cancer screening among ethnic minority groups in the UK

**DOI:** 10.1186/1471-2458-8-34

**Published:** 2008-01-25

**Authors:** Kathryn A Robb, Ijeoma Solarin, Emily Power, Wendy Atkin, Jane Wardle

**Affiliations:** 1 Cancer Research UK Health Behaviour Research Centre, Department of Epidemiology and Public Health, UCL, Gower Street, London, WC1E 6BT, UK; 2 Cancer Research UK Colorectal Cancer Unit, St. Mark's Hospital, NW London Hospitals Trust, Northwick Park, Watford Road, Harrow, Middlesex HA1 3UJ, UK

## Abstract

**Background:**

Colorectal screening by Flexible Sigmoidoscopy (FS) is under evaluation in the UK. Evidence from existing cancer screening programmes indicates lower participation among minority ethnic groups than the white-British population. To ensure equality of access, it is important to understand attitudes towards screening in all ethnic groups so that barriers to screening acceptance can be addressed.

**Methods:**

Open- and closed-ended questions on knowledge about colorectal cancer and attitudes to FS screening were added to Ethnibus™ – a monthly, nationwide survey of the main ethnic minority communities living in the UK (Indian, Pakistani, Bangladeshi, Caribbean, African, and Chinese). Interviews (n = 875) were conducted, face-to-face, by multilingual field-workers, including 125 interviews with white-British adults.

**Results:**

All respondents showed a notable lack of knowledge about causes of colorectal cancer, which was more pronounced in ethnic minority than white-British adults. Interest in FS screening was uniformly high (>60%), with more than 90% of those interested saying it would provide 'peace of mind'. The most frequently cited barrier to screening 'in your community' was embarrassment, particularly among ethnic minority groups.

**Conclusion:**

Educational materials should recognise that non-white groups may be less knowledgeable about colorectal cancer. The findings of the current study suggest that embarrassment may be a greater deterrent to participation to FS screening among ethnic minority groups, but this result requires exploration in further research.

## Background

Colorectal cancer is the second leading cancer killer in the UK [1]. Mounting evidence points to the potential for mortality reduction by screening for early stage colorectal cancers or pre-cancerous adenomas [2-4]. Several screening methods are in use world-wide, including faecal occult blood testing (FOBT), flexible sigmoidoscopy, colonoscopy, and barium enema. In the UK, the National Bowel Cancer Screening Programme (NBCSP) is implementing biennial faecal occult blood testing (FOBT) – a test that examines stool-samples for blood that may indicate abnormalities. Flexible sigmoidoscopy (FS), which involves direct visualization of the distal colon to detect and remove pre-cancerous growths with the aim of reducing incidence as well as mortality, is under evaluation [5].

In the UK FOBT pilot, the uptake rate was 57% [6], which is close to the rates found in randomised trials [7]. Participation in the UK FS Trial was considerably lower (39%) and comparable with other trials of FS [8,9], although this was likely to have been a consequence of the two-stage invitation procedure built into the trial design [10], in which potential participants were initially asked if they would be likely to attend and randomized only if they indicated they would attend. To assess the likely uptake rate of FS if offered as a service in a community setting, we recently undertook a feasibility study and found participation rates of 55% [11]. Although this initial estimate was based on a relatively small sample (N = 510), it suggests that acceptance of FS in the UK is likely to be similar to FOBT participation. For both types of screening, however, these uptake rates could mask considerably lower participation among some subgroups of the population.

The Race Relations (Amendment) Act (2000) [12] highlighted the need to ensure racial equality of opportunity to access services. Evidence from the cervical and breast screening programmes in the UK suggests lower participation among minority ethnic groups – particularly south Asians – than among the white-British population [13-15]. In a more recent analysis, Webb et al. [16] examined cervical screening records of 72,613 eligible women in Manchester. They found uptake in south Asian women to be 69.5%, compared with 73% in other women, although the differences diminished once area-level variables (deprivation, transience, social isolation) and practice-level variables (practice workload, structure and GP characteristics) were controlled. South Asians were also more likely to be 'never screened' than other women (14.7% vs. 10.3%), which was not explained by confounding effects. The UK FOBT Pilot also found lower uptake among south Asian groups, but were not able to assess uptake among African and Caribbean groups [17]. They concluded that further studies were urgently required to guide the development of strategies to achieve equitable uptake across all ethnic groups.

Assessing ethnic differences in uptake of cancer screening in the UK is difficult because of the lack of ethnic monitoring data collected at the Primary Care level [18]. Research studies have tended to use the name-recognition computer programme, *Nam Pehchan*, to classify people as either south Asian or non-South Asian [17]. The obvious limitation of this approach is that it only distinguishes south Asian groups, while the UK has many different ethnic communities with cultural beliefs about health that are not necessarily fully compatible with the dominant biomedical model [19]. There is therefore a need to explore culturally-specific beliefs and values about colorectal cancer and screening so that any future service could be offered equitably.

Previous research has indicated that non-white groups living in the UK perceive their risk of colorectal cancer as lower than the white population [20], suggesting that one explanation for lower participation in cancer screening could be lack of recognition of cancer risk. Objectively, there are ethnic differences in cancer risks [21-23], but rates in south Asians have increased in recent years – most likely because of lifestyle changes – while they have been falling for the rest of the population [24]. It is therefore no longer appropriate to consider them as 'low risk' for cancer.

The aim of this study was to understand beliefs about, and interest in, bowel cancer screening among ethnic minorities in the UK. Knowledge about colorectal cancer, attitudes to FS screening, and behavioural intentions regarding FS screening, were examined using both open- and closed-ended questions in men and women from the principal ethnic groups living in the UK. This is the first national survey of ethnic minority groups' views of colorectal cancer screening, and overcomes some of the limitations of studies relying on name recognition software.

## Methods

### Participants

Data were collected by adding questions to the January 2006 Ethnibus™ survey [25]. This is a monthly, nationwide omnibus tracking survey of adults aged over 16 years from the main ethnic minority communities living in the UK (Indian, Pakistani, Bangladeshi, African, Caribbean and Chinese). In total 750 interviews are conducted face-to-face by multilingual field workers each month, to which, for this study they added 125 interviews with white British people for comparison with the ethnic minority groups.

### Sampling

The Ethnibus™ survey uses quota sampling to obtain samples that are representative of the UK population in terms of the different ethnic groups. Using data from the 2001 Census, Ethnibus™ employs a random sampling process to identify sampling points (postal districts) for each of the main ethnic groups. The number of target interviews in each ethnic group is based on the national ethnic population proportions. Once sampling points have been selected (approximately 100–150), data on age and gender distributions for the sampling point are collated from the 2001 Census and used to determine age and gender quotas. Field workers then visit the selected postal districts and approach and screen households for eligibility. If the household contains an individual that meets the criteria of the quota then the individual will be invited to take part in a face-to-face interview. If they decline, the fieldworker moves on to the next household, and so on, until their quota is reached. Ethnibus report that interviews are carried out in 75 – 80% of households that have an eligible individual.

### Measures and procedures

As an introduction to the questions on colorectal cancer, participants were told: 'These are some questions about bowel cancer from Cancer Research UK.'

#### Perceived causes of colorectal cancer

Participants were first asked the open-ended question: 'What do you think are the main things that increase a person's chance of developing bowel cancer'. This item has previously been used in population surveys in the UK [26]. Participants were not prompted and responses were recorded verbatim.

#### Interest in colorectal screening

Brief information about the screening test was read out by the interviewer before the next question as follows: 'A new test may become available which helps to prevent bowel cancer. It works by removing growths which can turn into cancer if they are left. During the test the nurse inserts a thin flexible viewing tube into your back passage to look for growths. If the nurse finds any, they can be removed quickly and painlessly during the test. Removing any growths helps to prevent bowel cancer. The test would be free on the NHS, it would take only 5 minutes, and would be done in a hospital clinic'. This information was followed by the question: 'If you were invited to have the test, would you take up the offer'. Response options were: 'yes, definitely; yes, probably; probably not; definitely not', as used in the UK FS Trial [27].

#### Reason for level of interest in screening

The interest item was followed by the open-ended question: 'Can you say why you would [definitely/probably/probably not/definitely not] take up the offer'. Responses were recorded verbatim.

#### Views on barriers to screening among community

In order to circumvent difficulties associated with personal disclosure, participants were also asked the open-ended question: 'In your community, what sorts of things might put people off having a test to prevent bowel cancer'. Responses were recorded verbatim.

#### Sociodemographic characteristics

Ethnibus™ includes questions on: age (16–24 years; 25–34; 35–44; 45–54; 55+); gender; marital status (married/cohabiting; single; divorced/separated/widowed); ethnicity (Indian; Pakistani; Bangladeshi; Caribbean; African; Chinese; white British); main language spoken at home (English; Urdu; Punjabi; Gujarati; Sylheti; Cantonese; other); religion (Muslim; Christian; Hindu; Sikh; Confucian; Other); gross weekly income of the chief earner (<£100; £101–200; £201–300; £301–400; £401–500; £501–600; £601–700; £701+; don't know; no answer); employment (full-time; part-time; not working); and locality (London; Midlands; South; North). Information on socio-economic status (SES) used the National Readership Survey social grading system (AB: managerial/professional; C1: supervisory; C2: skilled manual; D: semi-skilled and unskilled manual; E: state pensioners, casual/lowest grade workers) which is based on the occupation of the chief income earner and is the system commonly used in market research and in opinion polls (e.g. MORI [28]).

### Analysis

All analyses were done using SPSS 13.0. Questions with defined response options were analysed using Chi-square tests to identify proportional differences, although for some questions there were too few counts per cell for detailed comparisons. Interactions were tested with ANOVA to see whether demographic factors moderated ethnicity differences in interest in screening. Multivariate analyses were carried out using logistic regression analysis.

The open-ended questions were analysed using content analysis to identify participants' responses by ethnic group. Responses to the three open-ended questions were reviewed by two researchers (KR and IS) and a coding scheme was developed following the recommendations of Krippendorff [29]. Responses to the three open-ended questions were coded independently by researchers IS and EP for 88 interviews (10% of all participants). Inter-rater agreement was 90%, and the Kappa inter-rater reliability statistic [30] was 0.82, which represents an acceptable level of agreement [31]. The remaining participants' responses were coded by IS.

Coding the open-ended questions led to the construction of several categories for each question. The greatest variation was in responses to the question on perceived causes of colorectal cancer. In total, 19 categories were created to cover all responses. In addition, the most frequent category, 'diet', was subdivided into a further 17 categories. For the open-ended question on reasons for interest in screening, responses were analysed in two groups based on whether participants responded positively (yes, definitely; yes, probably) or negatively (probably not; definitely not).

## Results

### Sociodemographic characteristics

A total of 875 interviews were conducted. Sociodemographic characteristics of the sample are presented in Table 1. There were no significant differences in the gender distribution across ethnic groups. Pakistani, Bangladeshi and African respondents tended to have a younger age profile, while Caribbean and white-British respondents were slightly older. White-British interviewees had the greatest proportion of respondents in the divorced/separated/widowed category. When marital status was coded as married vs. not married, the Caribbean, Bangladeshi and Indian respondents were significantly more likely to be married. The relationship between ethnic background and religion was as expected, with all Pakistanis and Bangladeshis being Muslim, while having a Christian faith was reported most often by Caribbeans, followed by Chinese and white-British. Most Indians (70%) were Hindu and just over half of Chinese respondents were Confucian. Geographically, white-British and Chinese respondents were distributed across the four localities while African and Bangladeshi respondents were most likely to live in London. Very few Indian, Pakistani, Caribbean or African respondents lived in the South. English was the main language spoken at home by all Caribbeans and white-British, and by over 80% of Indians and Africans. White-British respondents were the least likely to be in full-time employment or be categorised in the highest socio-economic group. Indian and Chinese respondents were most likely to be in full-time employment and to be in the highest socio-economic status group. Over 40% of Pakistani, African and white-British respondents reported a weekly income of less than £300.

**Table 1:** Sociodemographic characteristics

	Total (n = 875)	Indian (n = 234)	Pakistani (n = 166)	Bangladeshi (n = 63)	Caribbean (n = 126)	African (n = 108)	Chinese (n = 53)	white-British (n = 125)	Significance
Gender %									
Male (n = 429)	49.0	49.1	50.6	52.4	46.0	48.1	50.9	48.0	χ^2^(6, 875) = 1.07, *P *= 0.983
Female (n = 446)	51.0	50.9	49.4	47.6	54.0	51.9	49.1	52.0	
									
Age %									
16–24 (n = 201)	23.0	21.4	30.1	31.7	16.7	21.3	24.5	19.2	χ^2^(24, 875) = 46.6, *P *= 0.004
25–34 (n = 224)	25.6	24.4	27.7	31.7	19.0	33.3	24.5	22.4	
35–44 (n = 189)	21.6	20.5	16.3	17.5	26.2	28.7	22.6	21.6	
45–54 (n = 127)	14.5	16.2	12.7	9.5	12.7	10.2	17.0	20.8	
55+ (n = 134)	15.3	17.5	13.3	9.5	25.4	6.5	11.3	16.0	
									
Marital status %									
Married/cohabiting (n = 404)	46.2	51.7	46.4	52.4	54.8	38.9	41.5	32.0	n/a*
Single (n = 402)	45.9	44.9	48.8	42.9	37.3	54.6	56.6	42.4	
Divorced/separated/widowed (n = 69)	7.9	3.4	4.8	4.8	7.9	6.5	1.9	25.6	
									
Religion %									
Muslim (n = 266)	30.4	5.6	100	100	0.8	21.3	0	0	n/a*
Christian (n = 335)	38.3	1.3	0	0	99.2	78.7	34.0	83.2	
Hindu (n = 164)	18.7	70.1	0	0	0	0	0	0	
Sikh (n = 54)	6.2	23.1	0	0	0	0	0	0	
Confucian (n = 30)	3.4	0	0	0	0	0	56.6	0	
Other (n = 26)	3.0	0	0	0	0	0	9.4	16.8	
									
Main language spoken at home %									n/a*
English (n = 736)	84.1	84.5	69.3	71.4	100	89.8	56.6	100	
Urdu (n = 21)	1.5	0	7.8	0	0	0	0	0	
Punjabi (n = 67)	6.5	8.1	22.9	0	0	0	0	0	
Gujarati (n = 17)	2.1	7.3	0	0	0	0	0	0	
Sylheti (n = 18)	1.9	0	0	28.6	0	0	0	0	
Cantonese (n = 23)	2.6	0	0	0	0	0	43.4	0	
Other (n = 11)	1.3	0	0	0	0	10.2	0	0	
									
Locality %									
London (n = 365)	41.7	25.6	42.2	66.7	52.4	75.0	34.0	22.4	χ^2^(18, 875) = 228.0, *P *< 0.001
Midlands (n = 258)	29.5	42.3	34.3	9.5	31.0	9.3	15.1	31.2	
South (n = 258)	9.3	2.6	2.4	14.3	5.6	4.6	30.2	27.2	
North (n = 171)	19.5	29.5	21.1	9.5	11.1	11.1	20.8	19.2	
									
Employment status %									
Full-time (n = 428)	48.9	62.0	51.2	46.0	42.9	50.9	58.5	23.2	χ^2^(12, 875) = 71.9, *P *< 0.001
Part-time (n = 167)	19.1	10.3	13.9	22.2	23.8	18.5	13.2	39.2	
Not working (n = 280)	32.0	27.8	34.9	31.7	33.3	30.6	28.3	37.6	
									
Socio-economic category %									
AB Managerial/professional (n = 178)	20.3	25.2	23.5	19.0	14.3	19.4	24.5	12.8	χ^2^(24, 875) = 58.2, *P *< 0.001
C1 Supervisory (n = 242)	27.7	25.2	33.1	25.4	27.8	25.9	28.3	27.2	
C2 Skilled manual workers (n = 232)	26.5	30.3	25.3	14.3	30.2	20.4	26.4	28.8	
D Semi-skilled/unskilled manual workers (n = 143)	16.3	15.4	7.8	20.6	15.9	19.4	17.0	24.8	
E State pensioners/unemployed (n = 80)	9.1	3.8	10.2	20.6	11.9	14.8	3.8	6.4	
									
Weekly income %									
£300 or less (n = 339)	38.8	31.9	47.6	38.1	37.3	45.4	22.6	43.2	χ^2^(12, 873) = 34.3, *P *= 0.001
£301–£500 (n = 344)	39.4	47.8	36.1	41.3	35.7	33.3	34.0	38.4	
£501 or more (n = 190)	21.8	20.3	16.3	20.6	27.0	21.3	43.4	18.4	

* insufficient counts per cell to perform Chi-square test

### Perceived causes of colorectal cancer

The most common response to the question on perceived causes of colorectal cancer was *'don't know' *(Table 2), with around 40% of respondents saying they did not know or could not think of anything that would increase a person's chance of getting bowel cancer. Bangladeshi respondents were most likely to say *'don't know' *(65%), and white-British respondents were least likely (11%). When responses were recoded into *'don't know' *vs. all other responses there was a significant difference by ethnicity [χ^2^(18, 875) = 228.0, *P *< 0.001].

**Table 2:** Perceptions of the causes of colorectal cancer %

	Total (n = 875)	Indian (n = 234)	Pakistani (n = 166)	Bangladeshi (n = 63)	Caribbean (n = 126)	African (n = 108)	Chinese (n = 53)	white-British (n = 125)
Don't know (n = 348)	39.8	37.6	50.0	65.1	45.2	48.1	24.5	11.2
Diet (n = 274) – breakdown below	31.3	34.2	23.4	17.5	28.4	23.1	45.3	47.2
*Bad diet (fast food, fatty foods) (n = 114)*	13.0	13.7	7.2	3.2	10.1	12.0	24.6	24.8
*Low fruits/vegetables diet (n = 34)*	3.9	5.1	3.0	1.6	1.8	0.9	5.7	8.0
*Low fibre/roughage diet (n = 27)*	3.1	2.2	0.6	3.2	1.8	3.7	7.6	7.2
*Spicy foods (n = 24)*	2.7	3.9	3.6	3.2	3.7	0	3.8	0.8
*Meat (n = 20)*	2.3	3.4	2.4	0	4.6	2.8	0	0
*Water (n = 20)*	2.3	2.6	1.8	3.2	0.9	0.9	1.9	4.8
*Cholesterol (n = 8)*	0.9	1.3	1.8	0	1.8	0	0	0
*Refined foods (n = 7)*	0.8	1.3	1.2	1.6	0	0	1.9	0
*Milk/dairy products (n = 5)*	0.6	0.8	0.6	0	0.9	0.9	0	0
*Salt/sugar (n = 5)*	0.6	0	1.2	1.6	0	0.9	1.9	0
*Carbohydrates (n = 4)*	0.4	0	0	0	2.8	0	0	0.8
*Caffeine (n = 3)*	0.3	0.4	0	1.6	0.9	0	0	0
*Fish (n = 2)*	0.3	0	0	0	0.9	0	0	0.8
*Protein (n = 1)*	0.1	0.4	0	0	0	0	0	0
Lifestyle (alcohol, smoking, weight, exercise, hygiene) (n = 94)	10.7	7.7	9.6	1.6	10.4	11.2	5.7	22.4
Psychological factors (stress, depression) (n = 53)	6.0	7.3	4.2	3.2	10.3	6.5	7.5	2.4
Environmental factors (pollution, preservatives) (n = 37)	4.0	3.4	4.8	6.3	4.0	2.8	3.8	5.6
Health problems (n = 29)	3.3	4.2	2.4	3.2	4.0	3.7	1.9	1.6
Family history (n = 17)	1.9	1.3	3.0	0	1.6	0.9	1.9	4.0
Lack of knowledge/ignorance (n = 12)	1.3	1.7	0	1.6	0	0.9	0	4.8
Other (n = 9)	1.0	0.9	1.2	1.6	0.8	0.9	0	1.6
Drugs/medicines (n = 5)	0.6	0.4	0	0	0.8	1.9	0	0.8
Chance (n = 5)	0.6	0.4	0.6	0	0	0	0	2.4
Psychosocial factors (e.g. poverty, unemployment) (n = 4)	0.4	0.9	1.2	0	0	0	0	0

NB: counts do not add up to 100% in each column as some participants mentioned more than one factor. In the diet breakdown percentages are for the whole sample.

Of those identifying a cause, about half suggested that diet played a part in risk of colorectal cancer; citing over-consumption of fast-food, fatty food or having a bad diet (21%), low or inadequate intake of fruits/vegetables (6%), and low fibre/roughage (5%). When responses were recoded into 'diet' vs. any other response, white-British (47%) and Chinese (45%) respondents were the most likely and Bangladeshi respondents (18%) the least likely, to suggest diet as an explanation [χ^2^(6,875) = 32.9, *P *< 0.001].

Interestingly, after diet and lifestyle factors, the next most frequent response was 'psychological factors' including stress and depression. Psychological factors accounted for about 10% of the responses given by those who provided a reason, and 6% overall. There were too few counts per cell to examine differences across ethnic groups.

### Interest in bowel screening

Interest in screening (probably or definitely) was high (68% overall) across all ethnic groups and when response options were dichotomised into interested vs not interested, there were no significant ethnic differences. Using all four categories of screening interest, white-British respondents were the most likely to report being definitely interested, while Caribbean respondents were the most likely to say they were definitely not interested (see Table 3).

**Table 3:** Interest in colorectal screening

	Total (n = 875)	Indian (n = 234)	Pakistani (n = 166)	Bangladeshi (n = 63)	Caribbean (n = 126)	African (n = 108)	Chinese (n = 53)	white-British (n = 125)	Significance
Interest									
Yes, definitely (n = 80)	9.1	6.0	6.0	7.9	7.9	4.6	11.3	24.0	
Yes, probably (n = 519)	59.3	64.1	68.7	58.7	57.1	60.2	64.2	37.6	
Probably not (n = 138)	15.8	12.4	14.5	15.9	12.7	14.8	13.2	28.8	χ^2^(18, 875) = 79.0, *P *< 0.001
Definitely not (n = 138)	15.8	17.5	10.8	17.5	22.2	20.4	11.3	9.6	
									
Interest dichotomised									
Yes (n = 599)	68.5	70.1	74.7	66.7	65.1	64.8	75.5	61.6	χ^2^(6, 875) = 8.6, *P *= 0.195
No (n = 276)	31.5	29.9	25.3	33.3	34.9	35.2	24.5	38.4	
									
Interest in those aged 45+									
Yes (n = 167)	64.0	62.0	76.7	41.7	62.5	50.0	73.3	65.2	χ^2^(6, 261) = 7.9, *P *= 0.243
No (n = 94)	36.0	38.0	23.3	58.3	37.5	50.0	26.7	34.8	
									
Reasons for interest in screening (open-ended question) (n = 599)									
Peace of mind (n = 565)	94.3	95.1	91.1	97.6	93.9	91.4	95.0	98.7	
Important/can't ignore (n = 16)	2.3	3.7	3.2	2.4	1.2	4.3	2.5	.0	
Health maintenance (e.g. having a health check) (n = 14)	2.7	1.2	4.0	.0	3.7	4.3	2.5	.0	
Ease of having test (e.g. test sounds easy) (n = 4)	0.7	.0	1.6	.0	1.2	.0	.0	1.3	n/a*
									
Reasons for lack of interest in screening (open-ended question) (n = 276)									
Embarassment/shyness/shame (n = 68)	24.6	25.7	23.8	23.8	27.3	23.7	30.8	20.8	
Not interested (n = 36)	13.0	11.4	4.8	23.8	15.9	18.4	15.4	10.4	
Acceptability of method(n = 30)	10.9	4.3	11.9	4.8	11.4	18.4	15.4	14.6	
Other priorities (n = 24)	8.7	5.7	16.7	9.5	11.4	5.3	.0	8.3	
Pain(n = 19)	6.9	10.0	4.8	4.8	6.8	7.9	.0	6.3	
Fear (n = 17)	6.2	4.3	4.8	4.8	9.1	2.6	7.7	10.4	
No need/healthy/not necessary(n = 15)	5.4	7.1	4.8	.0	4.5	7.9	7.7	4.2	
Other (n = 14)	5.1	4.3	7.1	.0	6.8	2.6	7.7	6.3	
Values/culture/religion (n = 11)	4.0	5.7	7.1	4.8	.0	7.9	.0	.0	
Prefer alternative method(n = 10)	3.6	5.7	4.8	4.8	4.5	.0	.0	2.1	
Lack of time(n = 8)	2.9	.0	.0	9.5	.0	.0	.0	12.5	
Don't know/not sure (n = 8)	2.9	4.3	4.8	4.8	.0	2.6	7.7	.0	
Last resort (n = 6)	2.2	4.3	2.4	4.8	2.3	.0	.0	.0	
Need more info (n = 5)	1.8	4.3	.0	.0	.0	2.6	.0	2.1	
Don't want to think about it (n = 5)	1.8	2.9	2.4	.0	.0	.0	7.7	2.1	n/a*
									
Perceived barriers to screening uptake among community									
Embarassment/shame (n = 752)	85.9	97.0	97.0	100.0	95.2	100.0	96.2	17.6	
Nothing (n = 56)	6.4	.4	.6	.0	0.8	.0	1.9	41.6	
Logistics (time/place) (n = 30)	3.4	.0	.0	1.6	0.8	.0	.0	22.4	
Other priorities (n = 9)	1.0	.9	.0	.0	0	.0	.0	5.6	
Low level of awareness/interest (n = 9)	1.0	.0	.0	.0	0	.0	.0	7.2	
Pain (n = 9)	1.0	1.3	1.2	.0	1.6	.0	1.9	.8	
Culture/religion (n = 6)	0.7	.4	2.4	.0	0.8	.0	.0	.0	
Concerns about the test (n = 5)	0.6	.0	.0	.0	0.8	.0	.0	3.2	
Fear (n = 2)	0.2	.0	.0	.0	0	.0	.0	1.6	
Stigma (n = 2)	0.2	.4	.0	.0	0.8	.0	.0	.0	n/a*

*insufficient counts per cell to perform Chi-square test.

NB: counts do not add up to 100% in each column as some participants mentioned more than one factor

#### Age and interest in screening

Interest in screening among those older than 45 years (the group closest to the age for colorectal screening) did not differ by ethnic group (Table 3). Respondents under the age of 45 generally reported more interest in screening than older people (χ^2^(6,599) = 21.4, *P *= 0.002], but there was no interaction with ethnicity.

#### Marital status and interest in screening

There was a significant interaction between marital status and ethnic group [*F*(6,874) = 2.23, *P *= 0.038]. Being married was associated with greater interest in screening in all groups, but the effect was significantly stronger among white-British and African respondents.

#### Gender and interest in screening

Overall, significantly more men (72%) than women (66%) were interested in FS screening [χ^2^(1, 875) = 6.35, *P *= 0.012] with no significant ethnicity by gender interaction.

#### SES and interest in screening

**Figure 1:**
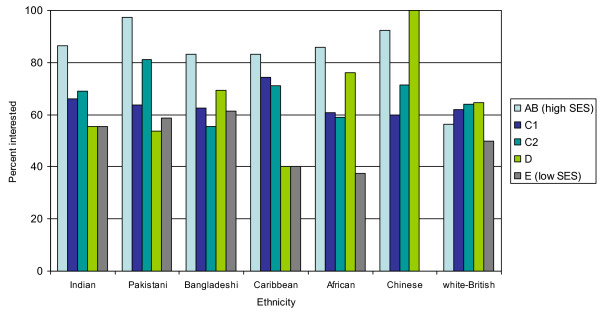
Interest in screening by SES.

The data indicated that across all ethnic groups, interest was higher in those at the managerial/professional level than in semi-skilled/unskilled jobs (Figure 1). There was no ethnicity by SES interaction.

#### Multivariate analysis

In a multivariate analysis, being male, younger (25–34 years), and in a higher SES group were the only variables that were significant predictors of interest in FS screening (Table 4). The effect of ethnicity was not significant.

**Table 4:** Predictors of interest in colorectal cancer screening

	Univariate Analysis		Multivariate Analysis	
	Odds ratio (CI)	*P*	Odds ratio (CI)	*P*
Ethnicity				
White-British	1.00		1.00	
Indian	1.46 (0.93, 2.31)	0.104	1.72 (0.38, 7.71)	0.481
Pakistani	**1.84 (1.11, 3.04)**	**0.017**	2.27 (0.76, 6.74)	0.140
Bangladeshi	1.25 (0.66, 2.36)	0.497	1.77 (0.55, 5.67)	0.336
African-Caribbean	1.16 (0.70, 1.94)	0.567	0.89 (0.49, 1.60)	0.692
African	1.15 (0.67, 1.96)	0.612	0.95 (0.49, 1.86)	0.891
Chinese	1.92 (0.93, 3.95)	0.077	2.30 (0.74, 7.16)	0.148
				
Religion				
Christian	1.00		1.00	
Muslim	1.19 (0.84, 1.69)	0.318	0.53 (0.21, 1.32)	0.171
Hindu	1.13 (0.76, 169)	0.798	0.50 (0.11, 2.15)	0.348
Sikh	1.18 (0.63, 2.20)	0.964	0.56 (0.12, 2.64)	0.463
Confucian	1.36 (0.59, 3.16)	0.761	0.48 (1.2, 1.92)	0.302
Other	**0.50 (0.22, 1.11)**	**0.034**	0.45 (0.18, 1.14)	0.093
				
Gender				
Female	1.00		1.00	
Male	**1.45 (1.09, 1.93)**	**0.012**	**1.49 (1.10, 2.02)**	**0.010**
				
Age				
55+	1.00		1.00	
45–54	1.12 (0.68, 1.86)	0.654	1.16 (0.68, 1.98)	0.586
35–44	1.14 (0.72, 1.80)	0.589	1.14 (0.70, 1.85)	0.604
25–34	**1.67 (1.05, 2.64)**	**0.030**	**1.66 (1.02, 2.72)**	**0.043**
16–24	1.47 (0.92, 2.34)	0.105	1.41 (0.86, 2.32)	0.174
				
Marital status				
Married	1.00		1.00	
Single	0.84 (0.62, 1.14)	0.263	0.82 (0.60, 1.12)	0.219
Separated/divorced/widowed	**0.46 (0.27, 0.77)**	**0.003**	0.58 (0.33, 1.02)	0.058
				
Socio-economic category				
AB Managerial/professional	1.00		1.00	
C1 Supervisory	**0.30 (0.18, 0.50)**	**0.001**	**0.30 (0.18, 0.49)**	**0.001**
C2 Skilled manual workers	**0.37 (0.22, 0.62)**	**0.001**	**0.36 (0.22, 0.62)**	**0.001**
D Semi-skilled/unskilled manual workers	**0.27 (0.16, 0.46)**	**0.001**	**0.29 (0.17, 0.50)**	**0.001**
E State pensioners/unemployed	**0.16 (0.09, 0.29)**	**0.001**	**0.16 (0.08, 0.29)**	**0.001**
				
Locality				
London	1.00		1.00	
Midlands	1.12 (0.79, 1.58)	0.541	1.10 (0.75, 1.62)	0.626
South	0.62 (0.38, 1.02)	0.058	0.64 (0.37, 1.13)	0.126
North	0.95 (0.64, 1.40)	0.779	0.92 (0.60, 1.41)	0.696

### Reasons for interest in screening

Respondents who said they were interested in screening provided reasons for their interest that fell into four broad categories (Table 3). By far the most common reason was that the test would provide *'peace of mind'*. This was cited by 94% (565/599) of interested respondents, and mentioned by at least 91% in all ethnic groups. Few other factors were mentioned. Even though the test was described as *preventing *bowel cancer, no-one cited this as a reason for their interest in screening. When responses were recoded into *'peace of mind' *vs. all other responses, there were no significant ethnic differences.

### Reasons for lack of interest in screening

The main reasons given by those who were not interested in screening were shame or embarrassment (25%: 68/275). For example one Caribbean man aged 44–55 said *'I will not turn up due to shame'*, and one Indian woman aged 55+ said '*too embarrassed talking about it with you, never mind the test'*, while a young Bangladeshi women said '*I tend to say yes, and then not turn up because of shame'*. The next most popular reason was 'no interest' (13%). One African man aged 25–34 years said he would '*never do this'*. The un-acceptability of the method was mentioned by 11%, with one Chinese man aged 25–34 years saying *'the tube seems off putting' *and an African woman aged 35–44 years saying *'people like me find this intrusive' *(Table 3). There was some evidence that embarrassment/shame was a greater deterrent to ethnic minorities, particularly Chinese and Caribbean, than white-British respondents. However when responses were recoded into those mentioning embarrassment/shame vs. other responses, there was no significant differences between ethnic groups. Bangladeshi and African respondents were more likely to state that they were simply *'not interested in screening'*. Responses coded broadly as 'values/culture/religion' only accounted for 4% of responses across the ethnic groups and were cited by 7% of Pakistani respondents and 8% of Africans, but not at all by Chinese, Caribbean or White-British respondents as reasons for not taking part in screening. Examples included: '*not my cultural way*' Pakistani male, aged 55+; '*not culturally right' *African female, 45–54 years.

### Perceived barriers to screening uptake 'in your community'

There was a striking disparity between responses given by the minority ethnic groups and white-British respondents for this item (Table 3). Overwhelmingly, the most frequently cited barrier to FS screening for '*others in your community*' was embarrassment/shame, and when responses were recoded into embarrassment/shame vs. all other responses the difference between ethnic groups was significant [χ^2^(6,875) = 543.0, *P *< 0.00]. This accounted for at least 95% of responses (Indian 97%, Pakistani 96%, Bangladeshi 98%, Caribbean 95%, African 100% and Chinese 96%). Examples included '*embarrassment' *(said by 752 respondents); one Indian man aged 55+ said *'it's a humiliation' *and a Caribbean women aged 55+ said *'older people will feel violated by this humiliation'*. In contrast, only 18% of white-British respondents mentioned embarrassment as a barrier for other people, and they were more likely to say there were no barriers (42%; *'Nothing' *white-British male, aged 45–54). Once again, culture/religion did not feature explicitly as an element in their responses concerning barriers to screening, and was only mentioned by 2.4% of Pakistanis and 0.4% of Indian respondents. Examples included: *'in our culture we don't do these kinds of tests unless vital' *said by a Pakistani woman aged 55+.

## Discussion

The present study examined awareness of the causes of colorectal cancer, and attitudes to and interest in FS screening, in men and women from the main ethnic groups living in the UK. The aim was to identify potential barriers to participation that might need to be addressed in equitable provision of FS screening. This was the first national survey to address interest in FS screening among people from a range of ethnic backgrounds including Indian, Pakistani, Bangladeshi, Caribbean, African, Chinese and white-British.

'*Don't know*' was the most frequent response to the question on what increased the chance of developing bowel cancer for all ethnic groups except white-British and Chinese. More than half (65%) of Bangladeshi, and 50% of Pakistani respondents, could not suggest a single cause of bowel cancer, compared with 11% of the white-British and 24% of Chinese respondents. It is possible that because white-British people have higher objective risk of cancer that they knew more people with the disease and were therefore more able to suggest causes. Indeed, a survey of breast cancer awareness found that 39% of white-British women reported that they had acquired knowledge about breast cancer from a friend or family member who had developed it compared with only 16% of women from ethnic minorities [32]. Nevertheless, it is a matter of concern that knowledge is so low in some ethnic minority groups.

Respondents who were able to offer a cause overwhelmingly proposed diet. This finding is similar to another UK national survey of predominantly white respondents that found that knowledge of the causes of bowel cancer was poor and the most frequent response was for dietary factors [26].

Expressed interest in FS (probably or definitely accept an invitation to screening) was extremely high (over 60%), with the majority of respondents from all ethnic groups citing *'peace of mind' *as the motivation. This concurs with work from the FOBT pilot which found no differences in initial willingness to be screened between UK south Asian and non-south Asian groups [17]. However, there were differences in uptake, suggesting that initial intentions were differentially translated into behaviour. Our finding that interest in FS screening does not vary by ethnic background is an encouraging first step, but it would be premature to conclude that actual attendance would either be as high as implied by this degree of interest, or equivalent across all ethnic groups, in view of the reported barriers.

Among all groups except Bangladeshis, there was a tendency for men to be more interested in FS screening than women, which corresponds with the UK FS Trial's finding of higher attendance in men [33]. Within most ethnic groups, higher SES individuals were more likely to express interest in screening than lower SES, as found in previous studies [34]. The differences within ethnic groups emphasise the importance of recognising that ethnic minorities are not homogeneous, but in the final multivariate analyses, SES rather than ethnicity was a significant determinant of interest.

Among respondents who said they were not interested, 'embarrassment' and 'shame' were the most frequently cited explanations, being mentioned by around a third of respondents although there were no differences by ethnic group. However, in contrast, when asked about barriers 'in your community', over 95% of the non-white respondents suggested 'embarrassment' as a reason for non-participation compared with only 18% of white-British respondents. This suggests that personal reasons for lack of interest in screening may not be a good reflection of prominent barriers to participation in the community, although the alternative is that sub-cultural stereotypes – including those held by members of the group – underestimate the community's enthusiasm for health promotion and disease prevention. Other studies with predominantly white samples have found embarrassment cited as a barrier to colorectal cancer screening [35,36], and it may have played a role in the lower levels of participation in FOB testing and follow-up colonoscopy among south Asians in the FOBT pilot.

There are limitations to this study. Ethnibus™ uses quota sampling and while the proportions of each ethnic group were representative of the UK, it seems unlikely that the people who were willing to participate in the survey are representative of those who may have declined the invitation to be interviewed. The age range of respondents was 16–55+ years and so only a minority of participants fell within the likely screening age range. However, in view of some older adults' dependence on children to translate mailed information, particularly in south Asians, as well as the issue of 'family decision making' [17], it is valuable to collect information from a wide range of ages. The question on barriers to bowel screening 'in your community' may have been interpreted differently by different ethnic groups depending on their interpretation of 'community'. We hypothesise that this question would have been most ambiguous for the white-British group because they tend to have a less strong sense of community.

We used an open-ended question to assess knowledge of the causes of colorectal cancer, and it is likely that prompting respondents by providing a list of possible responses would have shown higher levels of awareness [37]. However, we judged that so little is known about ethnicity and FS screening, it would be better to allow people to identify their own reasons and explain things in their own terms. In this way, we were able to solicit explanations that may otherwise have been missed.

The SES measure used in this study is based on a social grading system developed by the National Readership Survey, and it does not have the same theoretical underpinning as the National Statistics Socio-Economic Classification (NS-SEC; [38]). Nevertheless, it showed the same gradient in interest in FS screening as other measures of SES [34]. Finally, the survey only assessed interest in FS screening and not actual attendance.

## Conclusion

This is the first national survey of ethnic minority groups' views on bowel cancer screening. Despite high levels of interest and no differences between ethnic groups, there appears to be a general lack of knowledge about bowel cancer which was particularly pronounced among some ethnic minority groups. The results suggest that embarrassment may be a greater deterrent to participation among non-White groups, however further quantitative research is required to assess its impact on FS screening intention and behaviour because self-reported 'reasons for' doing or not doing a behaviour do not necessarily translate into significant predictors of action.

## Competing interests

The author(s) declare that they have no competing interests.

## Authors' contributions

KR, EP, WA and JW conceived and designed the study. KR and IS developed the coding scheme. IS coded and analysed the data. KR and IS wrote the first draft of the manuscript. All authors read and approved the final manuscript.

## Pre-publication history

The pre-publication history for this paper can be accessed here:


